# Crafting Informative Titles in Medical Articles to Enhance the Comprehension of the Study Findings

**DOI:** 10.31662/jmaj.2024-0023

**Published:** 2024-06-03

**Authors:** Shigeki Matsubara

**Affiliations:** 1 Department of Obstetrics and Gynecology, Jichi Medical University, Tochigi, Japan; 2 Department of Obstetrics and Gynecology, Koga Red Cross Hospital, Koga, Japan; 3 Medical Examination Center, Ibaraki Western Medical Center, Chikusei, Japan

**Keywords:** informative title, manuscript, paper writing, significance, title

## Abstract

Original articles in the medical literature should have informative titles, also referred to as declarative titles. A nondeclarative title expresses the study’s theme (topic) or, at most, the materials and methods used, whereas an informative title highlights the significance of the study findings (study’s significance) and, at the very least, its results. A manuscript is typically organized to cover (i) the theme, (ii) materials and methods, (iii) results, and (iv) conclusion (study’s significance). Consequently, a nondeclarative title typically encompasses only the (ii) stage, whereas an informative title extends to the (iii) or (iv) stages. This study underscores the importance of informative titles in medical papers and offers guidance for crafting titles that align with established paper writing fundamentals.

## 1. Embracing an Informative Title: A Proposal

The titles of original articles in the medical literature should be as informative as possible. In general, article titles are divided into three types: informative or declarative, descriptive, and interrogative (question) ^(1)^. The informative title is also referred to as the declarative title. In this paper, I use the term “informative title.” To denote titles that do not belong to the category of an “informative or declarative title,” I use “nondeclarative title” (instead of “noninformative title”) because, although all titles inherently convey some form of information, the term “noninformative title” may lead to misunderstanding.

A nondeclarative title should be avoided. A nondeclarative title indicates the study’s theme or problem, or at most, materials and methods (M&M), whereas an informative title covers not only the study theme and M&M but also the results. The title further expressing the study’s significance is the most informative and thus recommended. I explain this by contrasting it with paper writing fundamentals.

## 2. Adherence of a Manuscript to Paper Writing Fundamentals

If readers can quickly grasp a manuscript’s message, the manuscript is considered a good paper. To facilitate this process, the fundamentals of medical paper writing have been proposed ^(1), (2), (3)^, hereafter referred to as the “fundamentals.” Adherence of the title to the fundamentals is highly important because it serves as the initial point of contact for readers. On the basis of my experience of writing 533 PubMed-indexed papers and reviewing 2,501 manuscripts (Web of Science), I aim to provide guidance on crafting titles that align with the fundamentals.

## 3. Sentence Title, Interrogative (Question) Title, and Subtitle

Before delving into my narrative, I explore the three types of titles. Certain journals prohibit the use of an interrogative (question) or a sentence title. This restriction occurs because, in question titles, the message may not be readily understood, potentially leading to confusion, particularly with yes-no question titles ^(4)^. The sentence title typically uses the “present tense.” This section describes established facts that are usually not provided by a single study. We will not discuss the suitability of these two titles for use in medical papers. Regarding subtitles, many journals allow their use (excluding subsubtitles). Subtitles often articulate concepts effectively and are used in this manuscript.

## 4. Example: Manuscript on “Antibiotics Prevented Preterm Birth”

The following is a hypothetical abstract on the topic of preterm birth. I attempt to explain how to craft titles based on this scenario.

**Background (Introduction):** (i) Ascending lower genital tract infection can lead to intrauterine infection and uterine contractions, resulting in preterm birth. (ii) The effectiveness of eradicating genital tract bacteria in preventing preterm birth is unknown. (iii) We hypothesized that administering metronidazole to pregnant women during the midtrimester period could be a cost-effective measure for reducing preterm birth without adverse events.

**Materials and Methods:** Low-risk pregnant women participated in a double-blind randomized trial with metronidazole (+) (n = 500) versus metronidazole (-) (n = 500). The primary outcome was preterm birth incidence, and the secondary outcomes were adverse events and cost-effectiveness.

**Results:** Metronidazole (+) resulted in a significantly lower rate of preterm birth and required significantly lower costs than metronidazole (−). No adverse events were observed.

**Conclusion:** Routine midtrimester metronidazole administration is a novel strategy for effectively reducing preterm birth.

Please note that the introduction consists of three parts: (i) known, (ii) unknown, and (iii) problem (hypothesis or question), and in the conclusion section, the study’s significance is stated ^(2), (3)^. Let us consider four types of titles for this manuscript.

## 5. Titles Indicating Different Stages of the Manuscript: Four Categories

The key point of crafting a good title is to consider “to what extent the title expresses the information.” Table 1 illustrates the four types of titles based on their varying degrees of informativeness.

**Table 1.** Categories of Titles According to “To Which Extent Reflecting the Manuscript’s Content” .

1.	Type 1: A title reflecting only the theme (problem, hypothesis, or question)
	“Routine mid-trimester administration of metronidazole to reduce preterm birth”
	1’ (bottom) indicates the worst titles of this type.
2.	Type 2: A title reflecting the materials and methods (M&M)
	“Routine mid-trimester administration of metronidazole to reduce preterm birth: A double-blind study on 1000 pregnant women”
3.	Type 3: A title reflecting the results
	“Efficacy of routine mid-trimester administration of metronidazole to reduce preterm birth: A double-blind study on 1000 pregnant women”
	“Decrease of preterm birth rate in women with routine mid-trimester administration of metronidazole: A double-blind study on 1000 pregnant women”
4.	Type 4: A title reflecting the study’s significance
	“A new recommended strategy of routine mid-trimester metronidazole administration to effectively reduce preterm birth: A double-blind study on 1000 pregnant women”
	“A routine mid-trimester administration of metronidazole as a new recommended strategy to effectively reduce preterm birth: A double-blind study on 1000 pregnant women”
	
1’:	Extreme examples of the type 1 title, the worst titles.
	“Study on the relationship between routine mid-trimester administration of metronidazole and preterm birth” (“Research into” or “Analysis of”)
	“Prophylactical administration of antibiotics for preterm birth”

Types 1, 2, 3, and 4 reflect the theme, up to M&M, results, and study significance, respectively. The title is more informative in this order.

### 5-1. Titles indicating only the study’s theme

“Routine midtrimester administration of metronidazole to reduce preterm birth” simply indicates the preset study theme. Extreme examples are illustrated in 1’ in Table 1. The addition of phrases like “study on,” “analysis of,” or “research into” only increases the title length without contributing meaningful information. Avoid using these phrases.

### 5-2. Titles indicating up to M&M

In the type 2 title, “Routine midtrimester administration of metronidazole to reduce preterm birth: A double-blind study on 1000 pregnant women,” the addition of “a double-blind” and “1000 women” provides important information, making it superior to the first title. Readers will understand the theme + M&M.

### 5-3. Titles indicating up to the study results

In the type 3 title, “Decrease in preterm birth rate in women with routine midtrimester administration of metronidazole: A double-blind study on 1000 pregnant women,” “decrease,” thus the result, is added. Readers can understand that this treatment reduced the rate of preterm birth; however, they might raise concerns about a potential scenario in which this strategy causes severe anaphylaxis and incurs substantial costs, casting doubt on the recommendation of this strategy. This title still requires further improvement.

### 5-4. Titles indicating up to the study’s significance

Please consider the type 4 title, “A routine mid-trimester administration of metronidazole as a new recommended strategy to effectively reduce preterm birth: A double-blind study on 1000 pregnant women.” Although specific details regarding adverse events and cost-effectiveness (the secondary outcomes) are not explicitly provided, the phrase “as a new recommended strategy” indicates that the strategy also showed satisfactory results in these aspects. This type of title conveys the study’s significance.

The conclusion, and thus the significance, of this study is “Routine mid-trimester administration of metronidazole is recommended as a novel strategy to effectively reduce preterm birth.” The type 4 title, which is the best, describes the conclusion sentence. Convert the conclusion “sentence” into a “noun phrase”; then, you can craft the type 4 title. We will discuss this later using another study as an example.

## 6. Understanding the Relationship between Titles and Manuscript Structure

**Figure 1.**
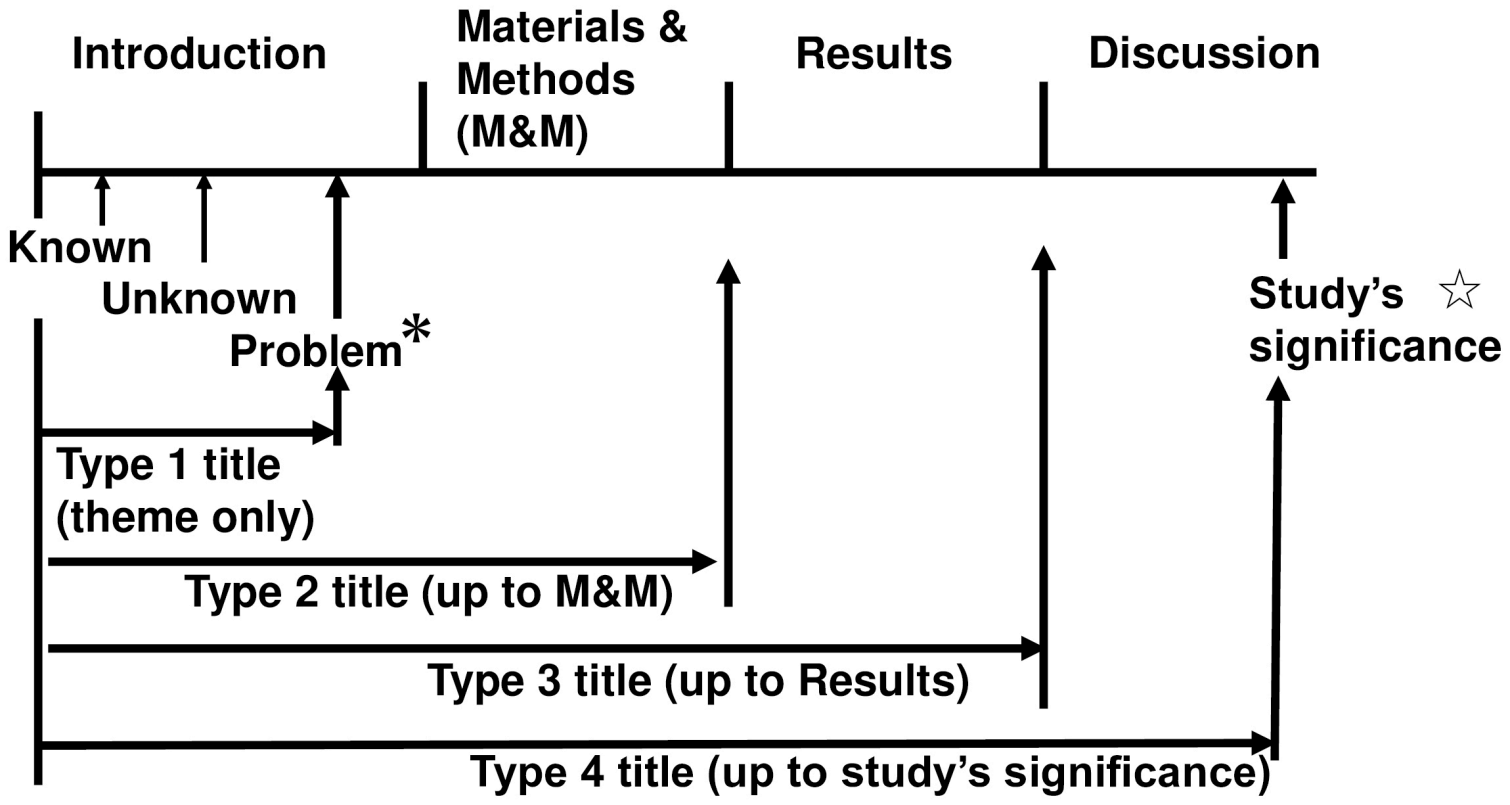
Schematic representation of the correlation between title types (1-4) and the degree to which each type reflects the content of the manuscript. The upper line illustrates the structure of the manuscript. *: The Introduction typically comprises three segments in the following order: known, unknown, and problem (alternatively termed hypothesis or question). The third segment, the problem (hypothesis or question), can be regarded as the “theme” of this study. ☆: The study’s significance is usually discussed toward the end of the Discussion section.

Figure 1 depicts the relationship between the four title types and the manuscript structure, highlighting the increasing informativeness of the titles. In the type 1 title, the readers can only understand the theme or problem. In the type 2, 3, and 4 titles, readers understand M&M, results, and study significance, respectively.

## 7. Challenges in Crafting Type 3 or 4 Titles

Very complicated data may prevent us from crafting a type 3 title, let alone a type 4 title. Consider a scenario in which the treatment strategy reduces the overall rate of preterm birth but increases that for births occurring before 28 weeks. A sentence title may be necessary, such as “A routine mid-trimester administration of metronidazole decreases the overall preterm rate but increases that of <28 weeks: A double-blind study on 1000 pregnant women.” Moreover, if some adverse events occur, preventing a conclusive recommendation regarding the treatment’s suitability, crafting a type 4 title, even in sentence form, becomes impossible. It is also worth noting that type 4 titles tend to be longer than other title types, and some journals may not favor lengthy titles. Striking a balance between informativeness and length is essential. Even when a “complete informative noun phrase title” cannot be crafted, the title should remain as informative as possible.

## 8. Application of the Technique to Other Medical Manuscripts

The proposed technique for generating informative titles is versatile and applicable to various medical topics. Table 2 presents a different scenario in which measuring X predicts the occurrence of disease Y, presenting a one-sentence expression and its corresponding title, which is aligned with title types 1, 2, 3, and 4.

**Table 2.** Examples of Creating a Noun Phrase Title from a Sentence Expression.

Type 1: Theme only:
We investigated whether measuring X predicted the occurrence of Y.
“Measuring X to predict the occurrence of Y”
Type 2: Up to Materials and Methods:
We investigated whether measuring X predicted the occurrence of Y using a new Z technique for detecting X.
“Measuring X to predict the occurrence of Y with the use of Z technique”
Type 3: Up to the Results:
A high X value predicted the occurrence of Y.
“Prediction of occurrence of Y by measurement of X using Z technique”
Type 4: Up to study’s significance:
A high X value predicted the occurrence of Y in the most effective manner among the reported methods.
“The best method to predict the occurrence of Y: measuring X using Z technique”

From types 2 to 4, some new words have been added: “with the use of Z technique,” “prediction,” and “the best method,” respectively.

## 9. Nondeclarative Titles Still Widely Used

The title of my inaugural PubMed paper in 1987 was ‘Ultracytochemical localizations of adenylate cyclase, guanylate cyclase, and cyclic 3',5'-nucleotide phosphodiesterase activity on the trophoblast in the human placenta: Direct histochemical evidence’ ^(5)^, which is a type 2 title (theme + M&M), indicating that I was not well-versed in the principles of crafting informative titles 3.5 decades ago. Interestingly, the situation does not seem to have changed significantly. As previously described ^(4)^, among the 44 original articles on COVID-19, 20 had informative titles, whereas 24 had nondeclarative titles.

## 10. Final Proposal: Thoughtful Consideration of Titles

Surprising or catchy titles capture readers’ attention and may be suitable for specific article categories, such as Perspectives, Views, or Poems (in JAMA). However, the titles of the original medical articles should not be surprising but should be as informative as possible. The current discussion focuses on the titles of original articles, with principles generally applicable to case reports and review articles.

Authors are encouraged to evaluate the informativeness of their titles by assessing the extent to which they accurately represent the content of a manuscript. A straightforward method to craft a type 4 title is to transform the conclusion sentence into a noun phrase. To the best of my knowledge, no experimental studies have confirmed the effectiveness of informative titles, and conducting such studies may pose challenges. Therefore, my proposal lacks supporting evidence. It is also important to note that differing opinions exist ^(1)^. Some argue that an informative (declarative) title may decrease readers’ inquisitiveness, and the inclusion of the results or conclusions in the title may seem presumptive and could become problematic if the findings are disproved. Thus, I do not claim that the proposed method is the ultimate solution. This is a personal perspective; however, I have used this approach for three decades, making it time tested. I believe that adopting this concept can help authors craft effective titles.

## Article Information

### Conflicts of Interest

None

### Acknowledgement

I thank Shinya Ito (University of Toronto, Canada), Daisuke Matsubara (Jichi Medical University, Japan), and Yuri Matsubara (Jichi Medical University, Japan) for their help.

### Auther Contribution

SM identified the significance and wrote the manuscript.

### Approval by Institutional Review Board (IRB)

Not applicable

### Patient Anonymity

Not applicable

### Informed Consent

Not applicable

### Data Availability

Data sharing is not applicable to this article because no new data were created or analyzed in this study.

## References

[1] Nundy S, Kakar A, Bhutta ZA. How to practice academic medicine and publish from developing countries?: a practical guide. Springer Nature Singapore Pte Ltd; 2022. Chapter 16, How to choose a title; p. 188-92.

[2] Matsubara S, Matsubara D. A checklist confirming whether a manuscript for submission adheres to the fundamentals of academic writing: A proposal. JMA J. 2024;7(2):276-8. 38721070 10.31662/jmaj.2023-0201 PMC11074514

[3] Matsubara S. How to write a good case report. Tokyo: Tokyo Medical Press; 2014. 286 p. Japanese.

[4] Matsubara S, Matsubara D, Matsubara T. Letter to 'Clinical update on COVID-19 in pregnancy: A review article': Obstetric or Gynecologic papers on COVID-19 should have an informative title. J Obstet Gynaecol Res. 2020;46(11):2455. 32924225 10.1111/jog.14470

[5] Matsubara S, Tamada T, Saito T. Ultracytochemical localizations of adenylate cyclase, guanylate cyclase and cyclic 3',5'-nucleotide phosphodiesterase activity on the trophoblast in the human placenta. Direct histochemical evidence. Histochemistry. 1987;87(6):505-9. 2891656 10.1007/BF00492464

